# Improving Time

**Published:** 1877-05

**Authors:** 


### *IMPROVING TIME.*

We are acquainted with a boy sixteen years old who is possessed of more useful information of a general character than is usually acquired by intelligent men at sixty years. He has gleaned most of it from "Zell's Encyclopedia," which, with "Webster's Unabridged Dictionary," he always keeps within reach while attending to his regular routine of duties, and to which he refers for information many times every day. We cannot imagine how any man who has a true appreciation of knowledge and a desire to promote the best interests of his children, can neglect to place these books within their reach. They may not at first be inclined to consult them, but their curiosity will soon induce them to do so, and this will be followed with a thirst for knowledge which may lead to the highest attainments.

